# Immunoglobulin Glycosylation – An Unexploited Potential for Immunomodulatory Strategies in Farm Animals

**DOI:** 10.3389/fimmu.2021.753294

**Published:** 2021-10-18

**Authors:** Kristina Zlatina, Sebastian P. Galuska

**Affiliations:** Institute of Reproductive Biology, Research Institute for Farm Animal Biology (FBN), Dummerstorf, Germany

**Keywords:** immunoglobulin, antibody, glycosylation, Fc receptor, pregnancy, lactation, vaccination

## Abstract

The function of antibodies, namely the identification and neutralization of pathogens, is mediated by their antigen binding site (Fab). In contrast, the subsequent signal transduction for activation of the immune system is mediated by the fragment crystallizable (Fc) region, which interacts with receptors or other components of the immune system, such as the complement system. This aspect of binding and interaction is more precise, readjusted by covalently attached glycan structures close to the hinge region of immunoglobulins (Ig). This fine-tuning of Ig and its actual state of knowledge is the topic of this review. It describes the function of glycosylation at Ig in general and the associated changes due to corresponding glycan structures. We discuss the functionality of IgG glycosylation during different physiological statuses, like aging, lactation and pathophysiological processes. Further, we point out what is known to date about Ig glycosylation in farm animals and how new achievements in vaccination may contribute to improved animal welfare.

## Introduction

**Figure 1:**
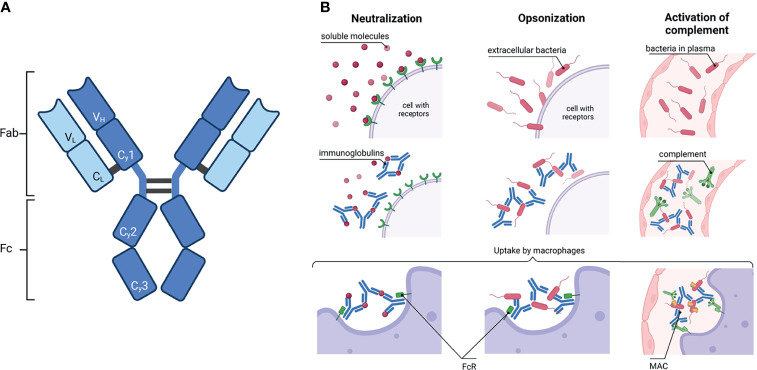
Immunoglobulin: schematic structure and how they activate the immune system. **(A)** A common IgG structure consisting of two heavy chain (dark blue) with three constant (Cγ1-3) and one variable domain (V_H_) and two light chain (light blue) with one constant (C_L_) and one variable domain (V_L_). The heavy and light chains are covalently connected by disulfide bonds. The IgG is further subdivided in the antigen binding fragment (Fab) and fragment crystallisable (Fc). **(B)** Activation of the immune system by antibodies. Left column: Ig can neutralize soluble molecules, e.g. bacteria toxins, to protect endogenous cells. Middle column: The binding of Ig to virus or bacteria antigens is called opsonisation. Right column: Ig bound to pathogens can activate the complement. Complement factors C1q recognize Ig and induce the complement cascade; a membrane attack complex (MAC) is formed in the end. In the end, macrophages or neutrophils phagocytose the complex of Ig with either soluble molecules or bacteria or, additionally, with the components of the complement. Created with BioRender.com.

Immunoglobulins (Ig) are essential players in the immune system. They recognize foreign molecules *via* their antigen binding sites, which are located in the variable domain of the antigen binding fragment (Fab) (**Figure 1A**). The recognition and binding of foreign molecules can induce several different defense strategies. For instance, soluble molecules such as toxins can be agglutinated and/or neutralized (**Figure 1B**). Furthermore, opsonization by Ig is an important process to counteract the invasion of pathogens. The recognition of antigens on the surface of pathogens subsequently initiate antibody-dependent cellular cytotoxicity (ADCC), antibody-dependent cellular phagocytosis (ADCP), or a complement-dependent cytotoxicity (CDC) (**Figure 1B**). Each of these three mechanisms is driven by an interaction of the fragment crystallizable (Fc) region with receptors of an effector cell or members of the complement system.

Remarkably, such interactions with the Fc region are influenced by its glycosylation status. For this reason, the detailed analysis of the glycosylation patterns of Igs during physiological and pathophysiological processes and the knowledge of the glycan-dependent functionality of Igs in mice and humans are increasingly being explored. However, very little is known about the glycosylation patterns of Igs in other mammals, such as farm animals. This is surprising given that an optimal functioning adaptive immune system is essential to ensure the health and welfare of animals.

This review gives a general overview of Ig glycosylation and its effect on the mechanisms of the adaptive immune system with the aim to demonstrate how Ig glycosylation has the potential to support the health and welfare of farm animals.

## Glycosylation of Ig

In eukaryotes, the majority of extracellular proteins is glycosylated (1). This post-translational modification of proteins is important to initiate cellular processes, such as recognition, communication, differentiating and binding events. Remarkably, the glycosylation status of proteins depends on several aspects. Firstly, within the animal kingdom, significant differences in the glycosylation machinery exist; for example, enzymes that are necessary for the synthesis and utilization of monosaccharides are species dependent, so that one and the same protein can be decorated with various glycan structures. Furthermore, the cell type, its differentiation state, and its metabolism status have an impact on the glycosylation patterns. Therefore, different physiological and pathological conditions frequently come with an altered glycosylation status. The most prominent forms of protein-glycosylations are the N- and the O-glycosylation.

**Figure 2:**
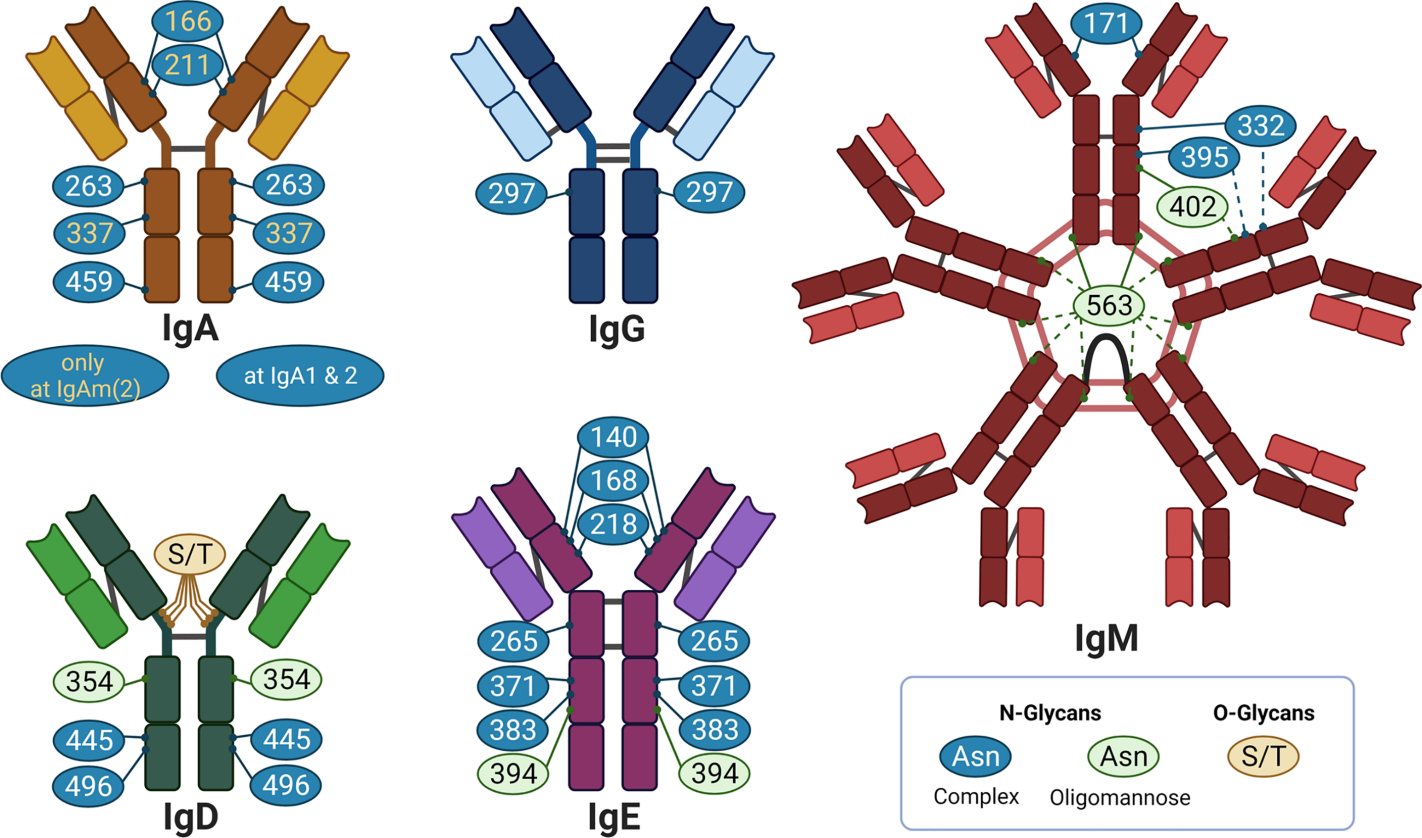
Overview of glycosylation sites of different Ig: IgA, IgG. IgM, IgD, and IgE in individual colors. The bright color illustrates the light chain, the dark color the heavy chain. The grey lines indicate disulphide bridges. Created with BioRender.com.

In the case of N-glycosylation, a precursor structure is co-translational transferred to an asparagine (Asn) residue of the nascent protein in the endoplasmic reticulum. The Asn must be part of the amino acid sequon Asn-X-Ser/Thr, whereby X can be any amino acid with the exception of proline. Thereafter, N-glycan processing starts, which includes numerous possible trimming and elongation events in the endoplasmic reticulum and Golgi apparatus (see **Supplemental Figure S1** for more information). Approximately 70% of all proteins carry one or more potential N-glycosylation sites (1). Further, Igs have several N-glycosylation sites (2). The number and positions differ between the individual Ig-classes and subtypes (**Figure 2**). For example, all IgG molecules are generally N-glycosylated in the Fc region at the conserved Asn_297_ (**Figure 2A**). Approximately 15-25% are additional N-glycosylated at the Fab region. However, no conserved N-glycosylation site exists in the Fab region (3). Altogether, N-glycans approximately account for 2-3% of their molecular weight. In contrast to IgG, the Ig-classes IgM, IgD and IgE contain significantly more conserved N*-*glycosylation sites and are much more N-glycosylated (~12-14% of the molecular weight) (2).

Besides N-glycans, Igs can also contain O-glycans (**Figure 2**). O-glycans are attached to the oxygen atom of serine (Ser) or threonine (Thr) residues. In contrast to N-glycosylation sites, no specific sequon exists and, thus, O-glycosylation sites are difficult to predict. To date, little is known about the impact of O-glycans on the functionality of Igs. For this reason, the review is focused on the structure and mode of action of N-glycans on Igs.

**Figure 3:**
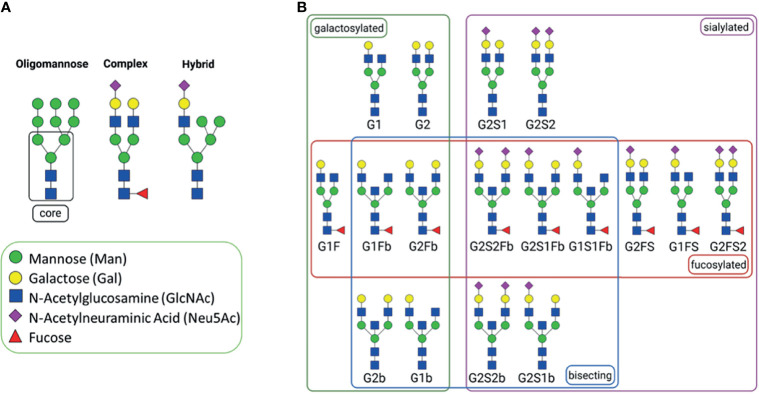
N-Glycans. **(A)** general types of N-glycans; all of them have a common core structure, highlighted with a square (1). **(B)** Commonly abundant glycans of human IgG, grouped by glycan affiliation. The N-glycans were created using GlycoWorkbench 2.1 (4) and arranged with BioRender.com.

Commonly, Igs are primarily decorated with complex N-glycans, but oligomannose and hybrid N-glycans can also be present (**Figure 3A**). In **Figure 3B**, for example, common N-glycans at Asn_297_ of human IgG are displayed, which frequently contain core fucose, bisecting N-acetylglucosamine (GlcNAc), galactose (Gal), and N-acetylneuraminic acid (Neu5Ac) residues. Oligomannose structures are mainly located in the Fab region of IgG (5). Overall, it appears that the heterogeneity of the glycan structures is higher in the Fab region (5).

**Figure 4:**
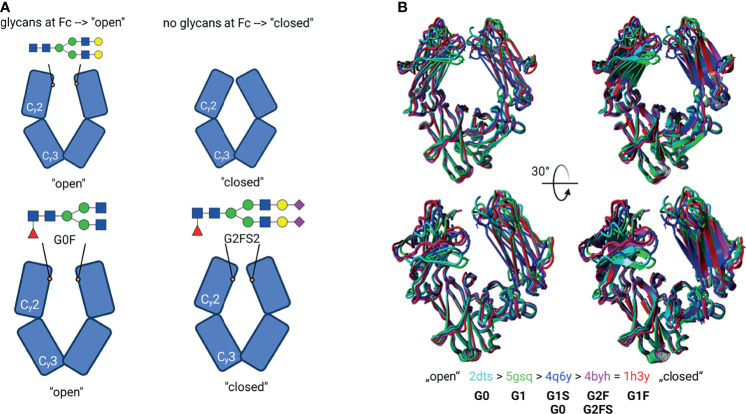
Glycosylation-induced conformational changes. **(A)** Schematic model of the IgG Fc structure with or without glycans. Top: Glycan structures by self-induce conformational changes, resulting in an “open” structure. The de-glycosylated Fc have a “closed” structure instead. Bottom: Suggested model for individual glycan-induced conformational changes. Different glycan structures are discussed to influence the distance between the Cγ2 domains. E.g., sialylated (G2FS2) glycans lead to a sterically closer conformation of Cγ2 to each other. Created with BioRender.com **(B)** Superpose of different Fc structures. The PDB entries correspond to the colour code in the cartoon- and tube-styled structures; additionally, their glycan structures are given. The structures were classified as “open” and “closed” conformation. The structures were superimposed using YASARA. The N-glycans were created using GlycoWorkbench 2.1 (4).

Glycans not only contribute to an altered molecular weight but also change the structural conformation (**Figure 4**). The presence of glycans at Asn_297_ within C_H_2 of Fc entails an “open” conformation (**Figure 4A** top), while enzymatic deglycosylation leads to a “closed” structure instead (6). Further, it is being explored whether distinct sugars, like sialic acid, induce additional conformational changes of the C_H_2 of Fc (7–9). Sondermann et al. described that the addition of sialic acid leads to a more “closed” Fc structure as compared to the presence of other glycan species or desialylated glycans (bottom in **Figure 4A**). Similar results were obtained by Ahmed et al. (8). In this review, the PDB structures used by Sondermann et al. and those generated by Ahmed et al. were aligned in order to compare the structural conformation. Five Fc regions are overlaid in **Figure 4B**, and their structures are related depending on the conformation between “open” and “closed”. Their glycans, which were present and obtained during the structural characterization, are given below the corresponding PDB entry. This comparison shows that the presence of sugar residues, like sialic acid and fucose, lead to closer Fc conformation. However, it has to be mentioned that, in fluids, molecules are flexible and glycans significantly increase structural variations, which cannot be completely reproduced when the crystal structures are obtained. Therefore, molecular dynamic tools have to be applied. Frank et al. analyzed Fc structures using such a strategy and observed high flexibility of both the glycans and the C_H_2 of Fc (10). Nevertheless, whether and to what extent different glycan patterns contribute not only to structural changes but also to the functionality of IgGs is under discussion.

### The Impact of Glycosylation on the Functionality of Igs

Among other mechanisms of the immune system, Igs mediate an immune response through the complement system. The classical pathway of the complement system is activated when the C1-complex molecule C1q binds to IgGs or IgM, which recognize an antigen on the cell membrane. The Ig/C1q complex induces a cascade of enzymatic reactions, leading to the formation of the membrane attack complex (MAC) and, thus, perforation of cellular membranes. Remarkably, the interaction with C1q is modulated by N-glycans of IgG_1_ at Asn_297_ (11). For instance, terminal galactose increases the binding to C1q (examples for different N-glycans are shown in **Figure 3A**). The observed effects might be the result of a changed 3D structure of the Fc region, as displayed in **Figure 4**. Thus, N-glycans seems to influence one of the key mechanisms of the classical complement pathway.

**Figure 5:**
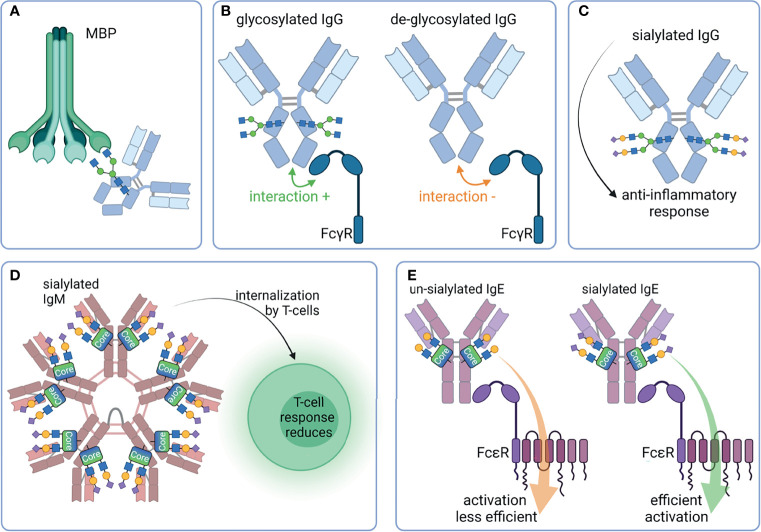
Overview of selected properties of Ig that are affected by glycosylation. **(A)** Mannan-binding protein (MBP) binds G0 glycan structures of IgG and induces the lectin complement pathway. **(B)** FcγR bind better to glycosylated Fc as compared to de-glycosylated Fc. **(C)** Sialylated IgG has an anti-inflammatory effect. **(D)** Sialylated IgM are internalized by T-cells, leading to a reduced T-cell response. **(E)** Un-sialylated IgE activates FcϵR less efficient than sialylated IgE. Created with BioRender.com.

Moreover, the lectin complement pathway can be induced if agalactosylated N-glycans are present on IgG. Typically, this pathway is initiated by the binding of mannan-binding protein (MBP) to oligomannose on the cell surface of pathogens. Via the MBP associated serine protease (MASP) an activation of the complement system is initiated. Malhotra et al. observed that agalactosyl N-glycans on IgG are also recognized by MBP, leading to an activation of the lectin complement pathway (12) (**Figure 5A**). Interestingly, these same glycan structures (mannose or GlcNAc residues) are also recognized by the mannose binding receptor (CD 206). This receptor is expressed in macrophages and dendritic cells (13). The binding of CD 206 to agalactosylated IgGs results in their uptake.

Further immunomodulatory interaction partners of Igs are their related Fc receptors (FcR). The major classes of Fc receptors are Fc-gamma receptors (FcγR), FcαR, FcϵR, and FcμR, which bind IgG, IgA, IgE, and IgM, respectively. FcμR recognize IgA molecules in addition to IgM, although with a lower affinity. Besides the extracellular Ig binding domain, Fc receptors may intracellularly have an activating or inhibitory motif, namely the immunoreceptor tyrosine-based activation motif (ITAM) (e.g. FcγRI, FcγRIIa, FcγRIIc, FcγRIIIa) or the immunoreceptor tyrosine-based inhibitory motif (ITIM) (e.g. FcγRIIb). Interestingly, the removal of N-glycans from the Fc region of IgG significantly reduces their binding to FcγR and, thus, their effector functions (14–16) (**Figure 5B**). However, further structural changes alter the IgG/FcγR interplay. For instance, removal of core fucose enhance the monocyte, macrophages, granulocyte, and natural killer cells mediated ADCC (17–19). Moreover, defucosylated IgGs have a higher affinity to FcγRIIIa, resulting in improved effector functions (18, 20, 21) (**Figure 5A**). FcγRIIIa and FcγRIIIb are also glycoproteins, and their N-glycans at Asn_162_ are involved in binding with IgG *via* an direct interaction with N-glycans of the IgG at the Fc Asn_297_ (22). It seems that a core fucose at the Fc Asn_297_ inhibit this glycan-glycan interaction and reduces the affinity toward FcγRIIIa (23).

Also, sialic acid plays a key role in FcR mediated signaling. Terminal α2,6-linked Neu5Ac has an anti-inflammatory function (24). The presence of Neu5Ac reduces the binding to activating FcγR, and this interaction subsequently leads to increased expression of inhibitory FcγRIIb (25, 26) (**Figure 5C**). It is under debate whether the interaction of FcγR is reduced to sialylated IgG and the binding of CD23/DC-SIGN to IgG is increased instead (27, 28). A recent study investigated the binding of human sialylated IgG to cells expressing CD23 or DC-SIGN (29). It could not verify the binding of Fc- or Fab-sialylated IgG to one of the proteins. Crispin et al. could also not verify the binding of IgG Fc to DC-SIGN (9, 30). The impact of sialylation was also investigated in the case of IgM. For instance, sialic acid residues on glycans of IgM induce its internalization in T cells and a subsequent suppression of T-cell responses (31). This effect could be counteracted by desialylation and might be the result of a reduced binding affinity of FcμR for asialylated IgM (**Figure 5D**). Furthermore, the activity of IgE is influenced by its sialylation status (32). This type of Ig plays a crucial role in type 1 hypersensitivity. After exposure to allergens, cross-linked IgE activate basophil and/or mast cells, leading to their activation and the release of inflammatory mediators, such as histamine (33). Interestingly, the amount of IgE present seems to be less important than the grade of IgE-sialylation to the extent of reaction (32). There are hints that the activation of FcεRI is less efficient when asialylated IgE is bound. However, the exact mechanism is still not fully understood (**Figure 5E**).

These examples demonstrate that the N-glycan structures of Igs significantly influence their immunomodulatory capacities, which explains the rapidly growing interest in glycan-mediated mechanisms in different areas of life sciences and medicine during the last decade.

### Aging

**Figure 6:**
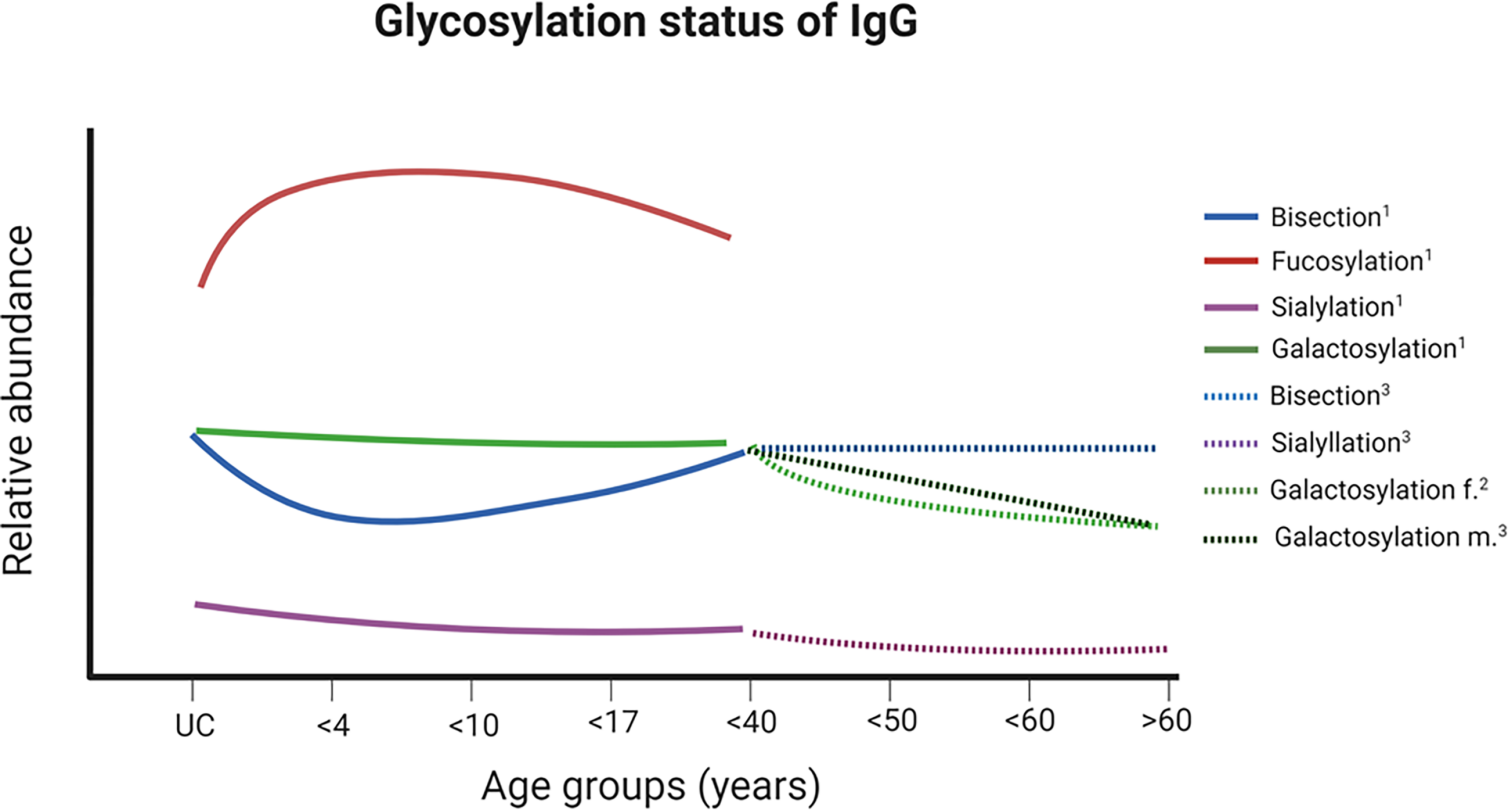
Changes of glycosylation status of IgG at Fc during ontogenesis. A schematic illustration of the relative ratios of various glycan structures and their changes over different ages are shown. For glycan structures examples and color codes, see **Figure 3B**. Further details are given in the main text. ^1^ (35), ² (36, 37), ³ (38). UC – samples from umbilical cord. Created with BioRender.com.

One of these scientific fields is aging, since the adaptive immune systems undergoes several changes during aging. Newborn mammals receive their first Igs from their mother. In humans and other mammals with a hemochorial placenta, such as rodents and primates, IgG can pass through the placenta barrier. As a consequence, offsprings are already equipped with IgG during pregnancy. Since, during pregnancy, IgG Fc N-glycans become significantly more galactosylated, sialylated, and less bisected (34), comparable glycosylation patterns can be observed in newborns (**Figure 6**). This glycosylation status of IgGs relates to the anti-inflammatory character of IgG (24). The increase of anti-inflammatory Igs might be necessary to suppress possible immune reactions between the unborn baby and the mother during pregnancy (35). When children begin to produce endogenous IgG, an altered glycosylation can be observed (**Figure 6**). Digalactosylated N-glycans decrease, but the level of monogalactosylated structures remains stable (35). Thus, in sum, the ratio of N-glycans with at least one galactose residue stays constant. In contrast, the status fucosylation and sialylation decreases and that of bisecting N-glycans increase (35). However, from the age of 40, the level of IgG galactosylation decreases (36, 39).

Moreover, the glycosylation of the Fab region was investigated in this context. Interestingly, during pregnancy, the glycosylation status of the IgG Fab region changes as well (5). These alterations were much more heterogeneous and different as compared to Fc glycosylation. For example, the percentage of monosialylated structures at Fc increases slightly during pregnancy and decreases after delivery. The opposite was observed at the Fab. Here, the monosialylated structures stay nearly at the same level during pregnancy and their amount increases after delivery. This fine-tuning of the glycosylation seems to regulate the effectiveness of IgGs during different physiological conditions, such as pregnancy or childhood.

### Ig Glycosylation Status During Lactation

As mentioned above, in species with a hemochorial placenta, such as rodents, primates, and humans, IgG can pass through the placenta barrier to equip offspring with Ig, whereas species with an epitheliochorial placenta, like ruminants, horses and pigs, are not able to transfer Ig *via* the umbilical cord. In these animals, the source of the first Igs is the colostrum. For instance, the gut of calves and piglets allows an unselective transition of proteins into blood circulation, approximately within the first 12-36 h postpartum. Calves and piglets that do not receive colostrum within the first 12 h have, for instance, reduced weight and increased mortality rates (40), and the administration of colostrum to calves within the first weeks of life reduces diarrheic disease (41). Thus, colostrum represents an essential source for Igs in farm animals, such as pigs and cows. However, Igs in matured milk also are important biomolecules to prevent pathogen invasion and to support the health of calves and piglets during their suckling period. This is also the case in species with a hemochorial placenta, such as humans. The major classes of Ig in milk are IgG, the secretory IgA (sIgA), and IgM. Their concentration varies during the lactation period (see **Table 1**) and is species and probably also breed-specific.

**Table 1:** Species dependent Ig amounts in colostrum and milk.

Species	Ig	Colostrum [mg/ml]	Mature milk [mg/ml]	Reference
*H. Sapiens* (human)	IgG	0.4	0.04	(42)
	IgM	0.3	0.03	(42)
	IgA	6-40	0.26-1.8	(42)
*B. taurus* (cattle)	IgG	15-180	0,5	(42)
	IgM	3-5	0.04	(42)
	IgA	1-6	0.05 -0.1	(42)
*O. aries* (sheep)	IgG	94-162	1	(43)
	IgM	1.3-21.2	0.2	(43)
	IgA	3.5	0.2	(43)
*E. ferus* (horse)	IgG1/IgG2 (IgGa)	82	0.2	(43)
	IgG4/IgG5 (IgGb)	183	0.3	(43)
	IgG3/IgG5 [IgG[T)]	44	0.1	(43)
	IgM	2.3	0.07	(43)
	IgA	9	0.7	(43)
*S. scofa d.* (pig)	IgG	618	1.6	(43)
	IgM	3.8	1.5	(43)
	IgA	11.3	4.3	(43)

**Figure 7:**
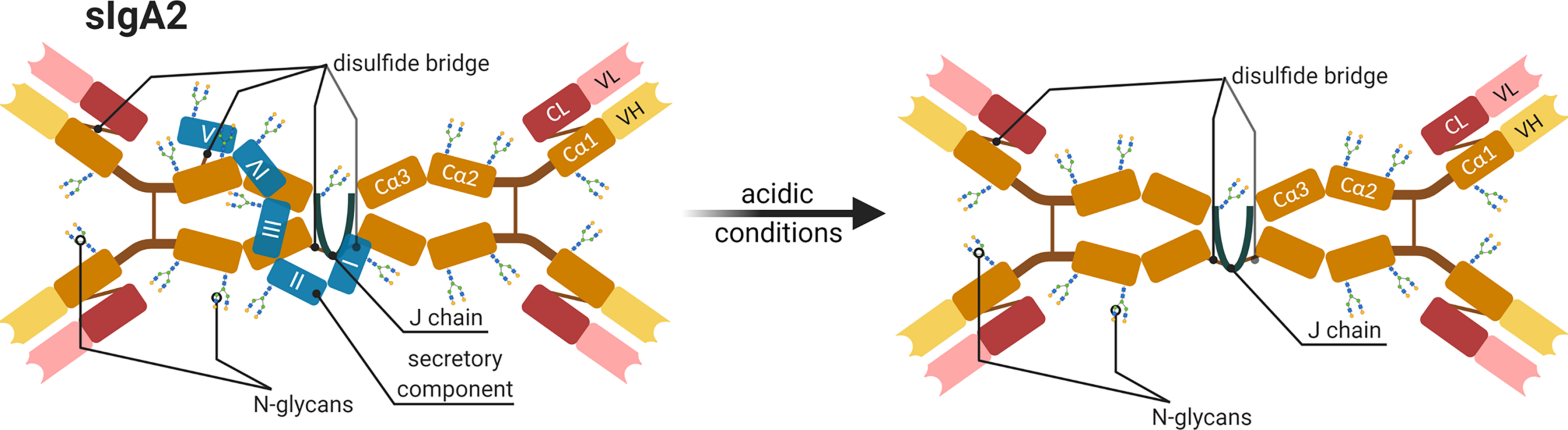
Schematic representation of a human secretory IgA subtype 2 (sIgA2). All components and possible glycosylation sites of sIGA2 as well as the changes occurring under acidic conditions are shown. The IgA dimer compose of four heavy chains (orange and yellow Cα1-3 and VH), four light chains (red and pastel-red), and a joining chain (J chain in dark blue) that connects dimers and a secretory component (blue I-V) by disulfide bridges (brown line) (45, 46). Created with BioRender.com.

There are several examples showing the importance of Ig glycans for the prevention of pathological bacteria in human. The members of the family Enterobacteriaceae, for instance, have Type 1 fimbriae on their surface with an adhesin that possesses mannose-specific lectin-like properties. This allows the bacteria to adhere to nasopharynx or colonic epithelial cells. After adhesion, they subsequently spread in the blood stream. The incubation of these bacteria with isolated secretory IgA (sIgA) from human milk leads to their agglutination, thus, their ability to bind to epithelial cells is inhibited (44). This is possible because glycans at the sIgA-Fc contain glycan structures with terminal mannose residues, which can bind using their fimbriae. **Figure 7** displays a schematic illustration of sIgA. Its specific joining chain (~16 kDa) connects the IgA dimers, and sIgA is associated with the secretory component (~75 kDa) that is additionally involved in the transport of IgA across epithelial cells. This transport is important to allow a transfer from the blood into milk. Usually, the secretory component shields these truncated glycan structures. However, acidic conditions in the digestive tract can disrupt the interaction between the secretory component and sIgA and enable the presentation of these sIgA glycans to bacteria (45) (**Figure 7**).

Furthermore, the S-fimbriated *E. coli* adhere to terminal Neu5Ac(α2,3)Gal at buccal epithelial cells and can induce neonate meningitis and sepsis. This interaction can be competitively inhibited by soluble glycans or glycoproteins, including sIgA from the human colostrum, which reduces the binding of *E. coli* to the host cells (47). The motif Neu5Ac(α2,3)Gal is also a common terminal structure of N-glycan on IgG. Thus, these antibodies might also act as competitors against bacterial adherence *via* their glycans. Moreover, Hanisch et al. demonstrated a strong interaction of S-fimbriae with N-glycolylneuraminic acid (Neu5Gc) (48). This sialic acid is usually not found in humans but in farm animals, like donkeys, cows, and pigs, and might prevent bacterial adherence and, thereby, an infection in their offspring.

Interestingly, a recent study detected Neu5Gc on glycans of sIgA in donkey milk (46). The milk sample was from an animal in the mid-lactating stage. They detected 5 N-glycosylation sites at the secretory component (N_83_LT, N_135_GT, N_291_QT, N_423_GT, and N_530_LT), two sites at the heavy chain of IgA (N_139_AS, N_338_VS [according to UniProt: N_134_AS, N_333_VS]), and one at the joining chain (N_72I_S). Furthermore, several O-glycans were present at the hinge region. The detected N-glycans were very heterogenic and included several fucosylated and sialylated structures with Neu5Ac as well as Neu5Gc. In addition, oligomannose N-glycans were present at the Fc region on Asn_291_ (46). It would be interesting if comparable inhibitory results can be achieved in relation to the adhesion of bacteria, as shown for sIgA from human milk. With the remarkable exception of Neu5Gc containing N-glycans, several of the glycan structures are comparable.

**Figure 8:**
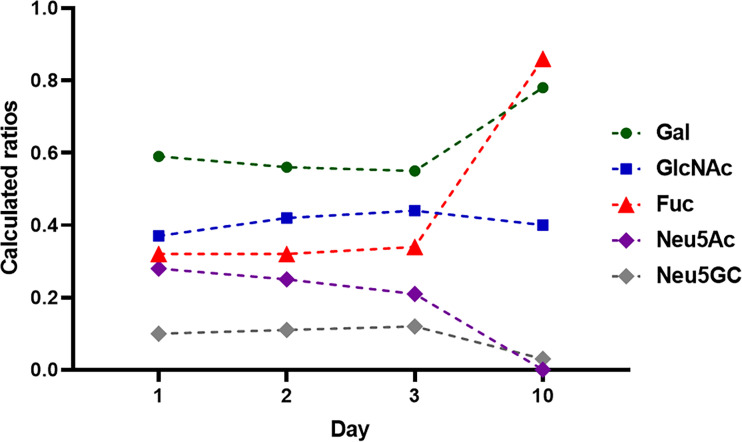
Monosaccharide composition during lactation from day 1-10. 1 mg IgG were analyzed at each time point. The monosaccharides ratios were calculated to 1 mol Man. Values are from (50).

In bovine milk, primarily, the glycosylation status of IgG was analyzed, representing the main Ig class in bovine milk (**Table 1**) (42, 43, 49, 50). In two studies, the composition of glycans from bovine milk at different time points of lactation period were examined. Feeney et al. investigated the IgG specific glycans by lectin-array assays at day 1, 2, 3, and 10 after birth (50). Takimori et al. determined the amount of IgG and its glycosylation status at the 1^st^ day and 1^st^, 2^nd^ 3^rd^ as well as 4^th^ week postpartum using MALDI-TOF MS (49). In this way, the short and longer term changes can be characterized. The biggest difference was observed in the sialylation of glycans (**Figure 8**) (50). The highest amount of sialylated glycans was detected in the colostrum, which rapidly decreases within the first three days (50). After 10 days, Neu5Ac is no longer detectable and only minor amounts of Neu5Gc are present. These results are in line with those of Takimori et al., who found that 50% of all IgG-glycans in the colostrum were sialylated. After 7 days, these sialylated structures were almost absent (49). Interestingly, the sialylated structures were only located at the Fc but not at the Fab fragment. Further, they investigated the glyosidic linkage between Neu5Ac and Gal using MALDI-TOF MS, which was in most cases α2,6-linkages. Feeney et al. used lectins to determine the linkage. The lectin MAA (from *Maackia amurensis*) detects mainly α2,3-linked Neu5Ac, whereas SNA (from *Sambucus nigra*) preferentially binds α2,6-linked Neu5Ac residues. The signal intensity using SNA indicated a higher abundance of α2,6-linked Neu5Ac residues, which is also in line with the results of Takimori et al. (49, 50) and suggest that, during first days of lactation, higher amounts of anti-inflammatory IgGs are present in bovine milk (24, 51, 52). In contrast to the sialylation status, the amounts of fucose and galactose residues at IgG stay constant within the first three days of lactation and slightly increase at day 10 (**Figure 8**) (50). GlcNAc and Man are stable throughout the first 10 days. The roles of individual glycan structures on IgG in milk are unknown. In Takimnori et al.’s study, the glycosylation status does not influence the binding to FcRn (49).

The results of Feeney et al. were obtained using the milk of Holstein Frisian cows representing high-performance dairy cows (50). The breed used by Takimori et al. was unfortunately not named. Breeds with lower level of milk production might have another glycosylation status during lactation. The few existing studies on buffalo milk could provide a first clue here. Two studies analyzed the glycan patterns of IgG in buffalo (*Bubalus bubalis*) milk at one single time point during lactation. The work of Bhanu et al. investigated a colostrum sample, and the work of Jineshet analyzed milk 14 days after parturition (53, 54). Bhanu et al. identified 54 different N-glycans, including oligomannose, neutral complex, and hybrid N-glycans, in addition to sialylated N-glycans (Neu5Ac and Neu5Gc). More than 20 of these glycans were fucosylated and the oligomannose N-glycans represented only a marginal ratio. However, the amount of sialylated N-glycans outweighed that of neutral structures. The high amount of sialylated and low amounts of fucosylated N-glycans are comparable to the results of Feeney et al. using bovine colostrum (50). The analysis of buffalo IgG glycans in mature milk (from day 10 of lactation) revealed the following distribution: 14% sialylated, 19% bisecting and 34% fucosylated glycans (54). However, in contrast to mature bovine milk, in which Neu5Ac was not detectable and only minor amounts of Neu5Gc were present, in buffalo milk, significant amounts of Neu5Ac and Neu5Gc were also detectable on day 14 of lactation (54). Thus, the dramatic loss of sialylated N-glycans does not take place in buffalo milk. Whether this glycosylation allows a broader panel of immunomodulatory mechanism is unknown so far. Nevertheless, the studies demonstrate that striking differences between various animal species exist.

## Relation Between IgG Glycosylation and Pathophysiology

Notably, the glycosylation patterns of Igs change also during pathophysiological processes. Previous studies indicate that glycosylation patterns alter during inflammatory processes like alloimmune thrombocytopenia (55) and active infections with pathogens such as HIV (56) or tuberculosis (57). On the other hand, changed glycosylation patterns can be detected in patients with chronic diseases like inflammatory bowl disease (58); autoimmune diseases like rheumatoid arthritis (RA) (59) and systemic lupus erythematosis (60); and neurological disorders such as multiple sclerosis (61, 62), Alzheimer’s disease (63), or Myasthenia gravis (64). In chronic diseases, it is mostly unknown where these changes come from and what their cause is. It is important to investigate whether pathological effects lead to an altered glycosylation or an altered glycosylation leads to pathological effects. In the latter case, it would be necessary to examine the cause for a changed glycosylation. This knowledge would be helpful to treat the diseases properly.

However, in some cases, such as RA, first insights have been described. About 0.5-1% percent of adults are affected by RA (65). Women are affected three times as often as men (66). Increased incidences of agalactosylated antibodies were earlier associated with higher disease activity (67). In 75-90% of patients, the disease activity reduces during pregnancy and increases again after delivery (68). As described above, pregnancy comes with changes in the glycosylation of antibodies. One study determining the glycosylation status during pregnancy revealed altered galactosylation and sialylation at the Fc of IgG (69). Furthermore, significant changes in bisecting N-glycans and fucosylation were observed for several IgG subclasses (69). Other studies have investigated the N-glycans of proteins from the whole serum and observed, for instance, decreased bisection N-glycans but an increase of galactosylation (70, 71). Reduced levels of pro-inflammatory bisection N-glycans, for instance, suggest an association with the reduced disease activity of RA patients during pregnancy.

In sum, a number of examples demonstrate that, in humans, sialylated N-glycans on IgG lead to immunosuppressive effects, whereas high levels of IgG without terminal sialic acid, galactose, and fucose residues but carrying bisecting N-glycans enhance inflammatory disorders and their severity. Thus, it is surprising that, with farm animals, studies investigating potential alteration in the glycosylation status of Ig during various physiological and pathophysiological processes are rare or completely missing so far. Knowledge about such glycan-dependent mechanisms might help develop novel strategies to increase welfare of farm animals.

### Vaccination Induced Immunization and Its Impact on Glycosylation of Ig

Since lack of terminal sialic acid, galactose, and fucose residues on N-glycans of the Fc region and higher amounts bisecting N-glycans increases the inflammatory capacity of IgGs, such glycosylation patterns are preferred after vaccination against pathogens (72). Bartsch et al. (73) recently examined how adjuvants influence the glycosylation of IgG with a special focus on sialic acid and galactose residues. In this study, mice were immunized for the first time with 100 µg ovalbumin (Ova) with different adjuvants and boosted second time with an Ova-PBS solution. The applied adjuvants included, among others, incomplete Freund adjuvant (IFA), complete Freund adjuvant (CFA), and alum. Interestingly, remarkable differences were found concerning the glycosylation of IgG. The application of eCFA (enriched CFA), IFA, and Montanide led to a significant reduction of galactosylation and sialylation. This effect was noticeably weaker when the adjuvants Alum, Adju-Phos, AddaVax, LPS, MPLA, R848, Poly (I:C) were applied. It should be noted that the stronger effectors - eCFA, IFA and Montanide - are “water in oil adjuvants”. The highest impact of all tested adjuvants were observed when eCFA was used. CFA was enriched with heat-killed *Mycobacterium tuberculosis* (Mtb). The effects might be mediated by the cord factor (glycolipid trehalose dimycolate) in Mtb extracts (57, 74). Whether comparable effects can be also achieved in farm animals is unknown.

### Glycoengineered Monoclonal Antibodies

Monoclonal antibodies (mAb) are an important and growing group of biotherapeutics for the treatment of cancer and chronic diseases. The Food and Drug Administration (FDA) approved more than 60 different mAbs and fusion molecules in the last few decades, which target in the most cases cancer cells (75). An optimized glycosylation has also already moved into the focus for therapeutic treatments with mAb. As mentioned above, the glycosylation status depends on many physiological conditions within an organism. Consequently, the glycosylation status is significantly influenced by the incubation and growing conditions of the IgG-producing cells and their genotype. To get a defined glycosylation pattern, the glycosylation-machinery of a cell lines can be manipulated using knock-out or knock-in strategies. For example, to obtain mAbs with nonfucosylated glycan structures, it is possible to knock out the α-1,6-fucosyltransferase (FUT8) (76) or overexpress β1,4-N-acetylglucosaminyltransferase III (GnTIII) (77). GnTIII catalyzes the addition of bisecting GlcNAc, which subsequently inhibits core-fucosylation. In both cases, the unfucosylated mAbs would enhance the ADCC, for example (17–19). Another option is to treat the mAbs *in vitro* using glycosidases. For instance, sialidases and galactosidases can be used to release sialic acid and galactose residues to obtain proinflammatory sets of antibodies (78). Consequently, the glycoengineering of therapeutic Abs is a powerful strategy to significantly improve their targeted application, such as with passive immunization.

### Avian Egg Yolk Antibodies (IgY)

**Figure 9:**
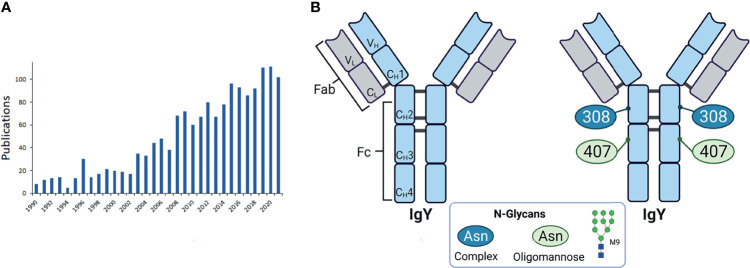
Avian egg yolk antibodies. **(A)** Entries of publications in PubMed with the keyword “immunoglobulin y” since 1990. **(B)** Structure of IgY. Shown are the domains of IgY with the two heavy chains (light blue) containing four constant domains (C_H_1-4) and one variable domain (V_H_), and two light chains (light grey) with one constant (C_L_) and one variable domain (V_L_). The heavy and light chains are covalently connected by disulfide bonds. The IgY is further subdivided in the antigen binding fragment (Fab) and fragment crystallisable (Fc). On the right the glycosylation sites are indicated with the detected glycan types. M9 is depicted as a representative of oligomannose type. Representative glycans for complex types are listed in **Table 2** and depicted in **Figure 3B**. Created with BioRender.com.

A further interesting type of antibody is the avian IgY. These Igs are of importance in various scientific fields. The number of publications listed on PubMed under the keyword “immunoglobulin y” increased steadily in the last years (see **Figure 9A**). Among others, it is gaining great interest for its application as a potential tool in diagnostic, therapeutic and biotechnology, since IgYs formed in poultry are very specific against mammalian proteins and have a high binding affinity. This is based on the phylogenetic distance between birds and mammals, thus, immunization works very well (79–82). The production of IgY is a further advantage. IgY can be easily isolated from the egg yolks of one and the same chicken. This is a non-invasive method and no blood has to be taken from the animals.

Compared to the mammalian IgG the avian IgY has one more constant domain in the heavy chain (CH3) and no hinge region. Further, there are two potential N-glycosylation sites. One at the CH2 and one at CH3 domain (83) (see **Figure 9B**). The analysis of the glycosylation status of IgY revealed mainly two types of N-glycans: oligomannose and complex type (see **Figure 3A**) with 37.2% and 62.8% respectively (83). Other studies detected also few hybrid glycans types at IgY (84, 85). The main structures, which were detected, in addition to their distribution are listed in **Table 2**. The CH3 domains contain only oligomannose structures, and the CH2 domain only complex-type structures (83). Further studies confirmed these results (85). For instance, Gilgunn et al. find on IgY originating from serum mainly complex, bi-, tri- and tetra-antennary glycans, which were partially bisected, fucosylated and sialylated. Besides these complex and high mannose structures, also hybrid structures were detected. Surprisingly, the impact of the glycosylation status on the activation of the host immune system and regulatory as well as signalling pathways have not been investigated so far and might represent a novel immunomodulatory tool improve animal welfare in poultry farming.

**Table 2:** Distribution of glycan types, calculated due to the molar basis of total N-glycans (83).

**Oligomannose type**	Monoglucosylated 26.8%	Others 10.5%	
**Complex type**	Neutral 29.9%	Monosialylated, 29.3%	Disialylated 3.7%
Structures corresponding **Figure 3B**	G1F; G1b; G1Fb; G2Fb	G1S1Fb, G2S1Fb, G2FS	G2S2Fb

## Conclusion and Outlook

The outlined importance of N-glycans for the structure of Igs and the resulting immunomodulatory capacities explain the rapidly growing interest in glycan-mediated mechanisms during the last decade and the application of highly defined glycoengineered Ig in human medicine (75–77). Thus, it is even more surprising that, in veterinary medicine, such studies are still limited or completely missed.

**Figure 10:**
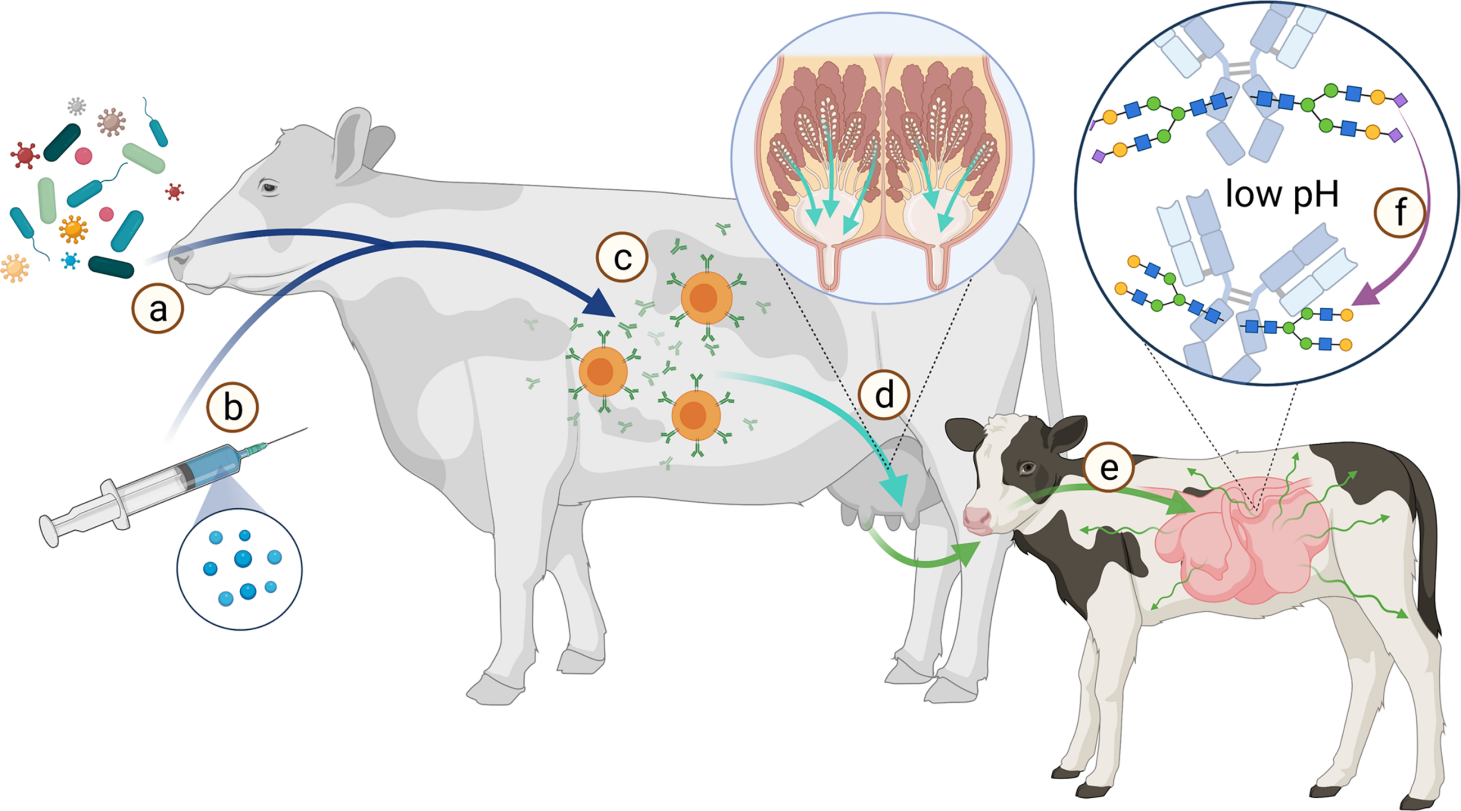
Scheme of the route from antigen to immunization of offspring. The immune system of the dam is stimulated by various pathogens **(A)** or vaccination **(B)** to produce antibodies **(C)**. These Abs are transported from the bloodstream into the milk **(D)**. During the first hours after birth, the calf’s intestine is permeable to proteins such as Abs **(E)**. This allows the calf to absorb Abs from the colostrum. Especially sialic acid residues (magenta diamond) are acid-sensitive and are cleaved from the glycans at low pH **(F)**. Created with BioRender.com.

It would be interesting to determine, whether feeding, social environment and vaccination have an impact on the Ig glycosylation status, since these factors influence the general metabolism of the animals. In addition, the knowledge of the relationship and influence of different adjuvants on vaccination success might increase the protection of farm animals against pathogens. Moreover, in the case of maternal vaccination, the offspring would be better protected during lactation, when milk IgGs contain optimized glycosylation patterns. The offspring of species with a hemochorial placenta, like humans, primates and rodents benefit during the pregnancy by a passive immunization, because here IgG can pass the blood-placenta barrier and protect the offspring. Species with an epitheliochorial placenta, like ruminants, including cattle, pigs, goat and sheep, but also horses, whales, and lower primates are not able to immunize their offspring passively through the placenta. Newborns of these species especially depends on a passive immunization from milk. Therefore, it would be interesting to examine if the glycosylation pattern of Ig changes during its transport from dams blood into the milk, and further through the stomach and gut of the offspring, until it reaches the blood system (**Figure 10**). This aspect is under present investigation in our lab. The knowledge about this could promote the development of vaccines and adjuvants to shift glycan structures to increase the efficiency of the immunisation. It would also promote the development and application of Ig with specific glycan patterns. In conventional farming it is common that calves are not suckled by their mothers. The colostrum given is usually from a colostrum pool, frozen colostrum or commercially available colostrum powder. At this point, glycoengineered antibodies could be additionally supplemented.

In sum, farm animals would benefit from a more detailed knowledge about all these aspects. Furthermore, animals in zoological gardens or species whose population sizes are small could have better chances of survival for their offspring. Thus, we propose that the glycosylation of Abs might represent a powerful target or tool to develop novel strategies to support the health and welfare of animals.

## Author Contributions

KZ and SG wrote the manuscript and gave the approval to the final version of the manuscript. All authors contributed to the article and approved the submitted version.

## Funding

The publication of this article was funded by the Open Access Fund of the Research Institute for Farm Animal Biology (FBN).

## Conflict of Interest

The authors declare that the research was conducted in the absence of any commercial or financial relationships that could be construed as a potential conflict of interest.

## Publisher’s Note

All claims expressed in this article are solely those of the authors and do not necessarily represent those of their affiliated organizations, or those of the publisher, the editors and the reviewers. Any product that may be evaluated in this article, or claim that may be made by its manufacturer, is not guaranteed or endorsed by the publisher.
