# Supplementary material for: Immunoglobulin Glycosylation – An Unexploited Potential for Immunomodulatory Strategies in Farm Animals
Source: Front Immunol. 2021 Oct 18;12:753294. doi: 10.3389/fimmu.2021.753294 (PMC8558360; doi:10.3389/fimmu.2021.753294)
Supplement: Supplementary file 1 [file DataSheet_1.pdf]

Supplementary Material

1 Supplementary Figures

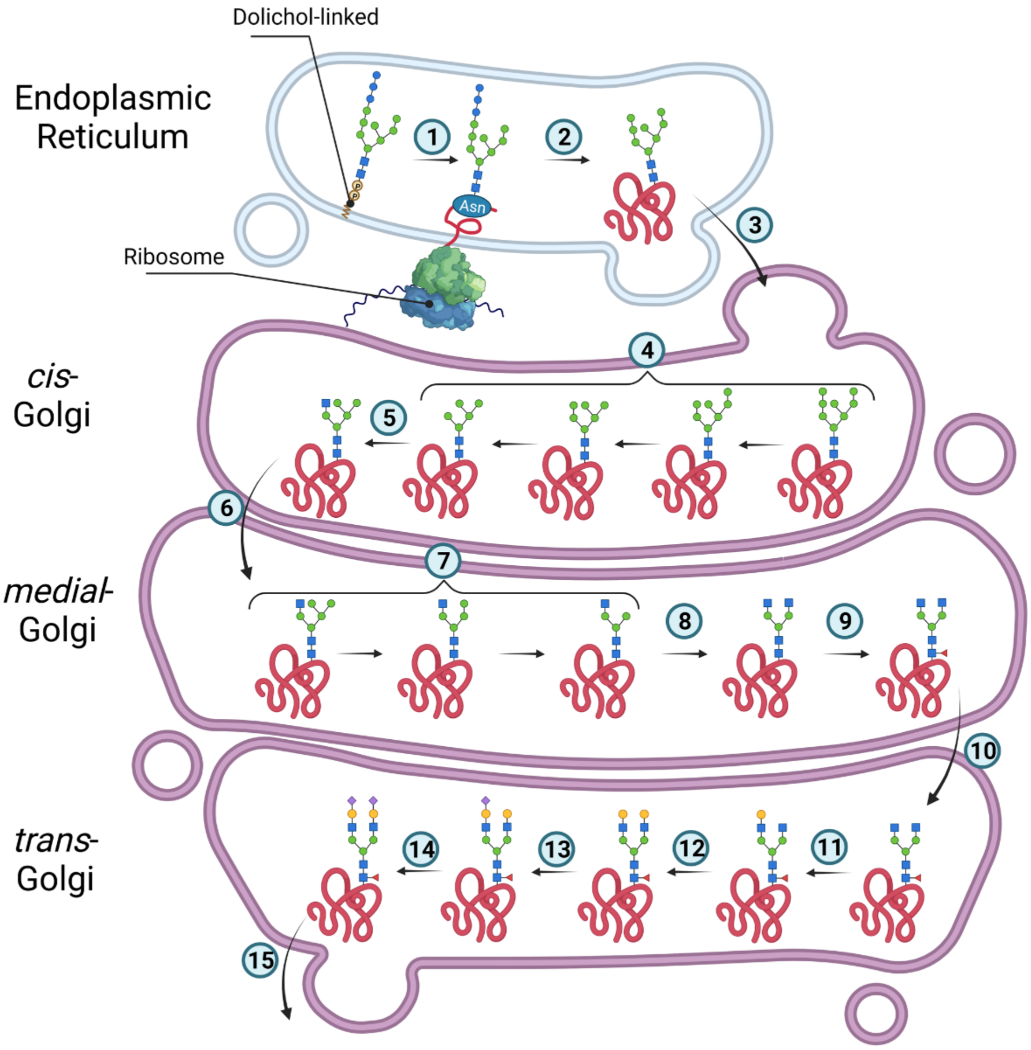

Supplementary Figure 1. Schematic illustration of the synthesis of a mammalian disialylated diantennary N-glycan. (1) In the endoplasmic reticulum (ER), the oligosaccharyltransferase (OST) co-translationally catalyze the transfer of the N-glycan precursor from dolichol to the carboxamide side chain of Asn within the nascent protein. (2) During protein folding and quality control, glucosidases trim the N-glycan. (3) Subsequently, the glycoprotein is vesicularly transferred to the cis-Golgi apparatus. (4) The trimming of the Man residues continues (5) and an N-acetylglucosamine (GlcNAc) is added. (6) Following that, the glycoprotein is transferred to the medial- Golgi. (7) Here, mannosidases release two more Man residues, (8) a further GlcNAc is added, and (9) a core fucose can be added as well. (10) In the trans-Golgi, the conversion of N-glycans continues with (11 and 12) the addition of galactose (Gal). (13 and 14) Finally, sialic acid is transferred to the diantennary N-glycan

and (15) the mature glycoprotein is transported to the cellular membrane. For more detailed information, please see (1, 2). The figure legend is based on (3). Created with BioRender.com.

2 REFERENCES

1. Stanley P, Taniguchi N, Aebi M. *Essentials of Glycobiology: N-Glycans*. Cold Spring Harbor (NY) (2015).
2. Chung S, Quarmby V, Gao X, Ying Y, Lin L, Reed C, et al. Quantitative evaluation of fucose reducing effects in a humanized antibody on Fcγ receptor binding and antibody-dependent cell-mediated cytotoxicity activities. *MAbs* (2012) **4**:326–40. doi:10.4161/mabs.19941
3. Zlatina K, Galuska SP. The N-glycans of lactoferrin: more than just a sweet decoration. *Biochem Cell Biol* (2021) **99**:117–27. doi:10.1139/bcb-2020-0106
